# Heterogeneity of ecological patterns, processes, and funding of marine manipulative field experiments conducted in Southeastern Pacific coastal ecosystems

**DOI:** 10.1002/ece3.4371

**Published:** 2018-07-25

**Authors:** Moisés A. Aguilera, Johanne Dobringer, Ignacio J. Petit

**Affiliations:** ^1^ Departamento de Biología Marina Facultad de Ciencias del Mar Universidad Católica del Norte Coquimbo Chile; ^2^ Programa Doctorado en Biología y Ecología Aplicada (BEA) Centro de Estudios Avanzados en Zonas Áridas (CEAZA) Universidad Católica del Norte Universidad de La Serena Coquimbo Chile; ^3^ Millennium Nucleus for Ecology and Sustainable Management of Oceanic Islands (ESMOI) Coquimbo Chile

**Keywords:** biotic interactions, coastal ecosystems, experimental complexity, marine experiments, Southeastern Pacific

## Abstract

Ecological manipulative experiments conducted in marine coastal ecosystems have substantially improved ecological theory during the last decades and have provided useful knowledge for the management and conservation of coastal ecosystems. Although different studies report global trends in ecological patterns worldwide, Southeastern Pacific coastal ecosystems have been poorly considered. Given that the SE Pacific coast encompasses diverse coastal ecosystems, consideration of studies conducted along this range can shed light on the heterogeneity of processes regulating coastal communities. We reviewed the biotic interactions and habitat type considered, as well as the complexity in terms of spatial and temporal extent of manipulative field experimental studies conducted along the SE Pacific coast from 0°S to 56°S (Ecuador to Chile). We test the effect of funding reported by different studies as a main factor limiting experimental complexity. From field ecological studies published from 1970 to 2016, we found that 81 studies were truly manipulative, in which one or multiple factors were “manipulated.” Around 77% of these studies were located between 21°S and 40°S, and conducted in intertidal rocky habitats. An increase in experimental studies was observed between 2010 and 2015, especially focused on herbivore–alga interactions, although we found that both the temporal extent and spatial extent of these studies have shown a decrease in recent decades. Funding grant amount reported had a positive effect on elapsed time of field experiments, but no effect was observed on spatial extent or in the biotic interactions considered. Elapsed time of experiments was different among the main biotic interactions considered, that is, herbivory, predation, and competition. We suggest that to further progress in applied ecological knowledge, it will be necessary to consider pollution and urbanization processes explicitly using a field experimental framework. This information could improve our understanding of how ecosystems present along the SE Pacific coast respond to climate change and increased levels of human interventions.

## 1 INTRODUCTION

Ecological experiments conducted in marine coastal ecosystems have been instrumental to the development of marine community ecology (Bertness et al., 2014). Because of this, manipulative experiments have experienced a strong increase during the last 40 years, being considered the most rigorous and persuasive tool of hypothesis testing in ecology (Raffaelli & Moller, 1999; Underwood, 2000). Manipulative field experiments in particular have provided important insight on the complexity and spatial scale of species interactions (Jenkins & Uyà, 2016; Witman, Lamb, & Byrnes, 2015). In operative terms, the complexity of manipulative experiments has been defined as the number of species, factors, and treatment combinations of an experiment performed (see Witman et al., 2015). The importance of complexity and the spatial scales considered in field experiments are considered critical to understand the dynamics of diverse coastal ecosystems. Comparative field experimental research could be designed to determine the levels of climate and human‐induced impacts on species interactions, biodiversity, and ecosystem functioning. A clear definition of patterns and processes included in hypotheses should be appropriate to the scale and complexity levels of an experimental study (Benedetti‐Cecchi et al., 2012; Underwood, 1999; Underwood & Petraitis, 1993). Development of field experimentation has been important in some places like North America, Australia, Europe (Witman et al., 2015), and apparently poor in South American coasts, which could limit the global understanding of ecological patterns and associated processes that regulate coastal ecosystems.

Previous specific reviews suggest that most marine ecological studies are concentrated in the SE Pacific coast (e.g., from 18° to 56°S), especially focused on herbivore–alga interaction (see Aguilera, 2011; Santelices, 1985; Vasquez & Buschmann, 1997). Although different coastal ecosystems across the Humboldt Current System are strongly interconnected by large‐scale processes like “El Niño” events (Tarazona & Arntz, 2001; Thiel, Thiel et al., 2007; Vinueza, Menge, Ruiz, & Palacios, 2014), there is little integrative work dealing with the different processes regulating coastal ecosystems in the SE Pacific coast. Knowledge of the spatial and temporal extent of field experimental studies as well as the biotic interactions considered in each study may be of interest in this context.

Strong emphasis on experimental manipulations and null hypothesis testing, particularly among rocky shore ecologists (Camus & Lima, 1995; Underwood, 1999), has been complementary to quantitative monitoring of biological systems, especially in Chilean coasts (Castilla, 2000; Moreno, 2001; Navarrete, Gelcich, & Castilla, 2010). Important insights from field experiments in Chile have resulted in applied knowledge to manage ecosystems under intense human harvesting and fisheries (Castilla, 2000; Fernández et al., 2000; Gelcich et al., 2010), and have also provided important attempts to summarize ecological knowledge in Chile (Castilla, 2000; Gelcich et al., 2012; Navarrete et al., 2010).

One important concern is the future of the field experiments in terms of incorporation of human impacts, coastal urbanization and pollution effects on species interactions, and coastal ecosystem structure (see Bulleri & Chapman, 2010; Firth et al., 2016; Johnston & Mayer‐Pinto, 2015; Johnston, Mayer‐Pinto, & Crowe, 2015 for discussions on this topic). There is no information, however, related to the number of field experiments conducted along the SE Pacific coasts which deal with coastal pollution and/or hard artificial infrastructures as reported for other coasts (Strain et al., 2017). The temporal and spatial scales of experimental studies are critical to detect strong versus weak natural/anthropogenic impacts as well as to discriminate between negative versus positive species interactions in aquatic ecosystems (Stachowicz, Best, Bracken, & Graham, 2008; Stachowicz, Bruno, & Duffy, 2007). Incorporation of larger spatial and temporal scales in experimental designs could successfully account for the heterogeneity of natural ecosystem processes and levels of human interventions (Jenkins & Uyà, 2016; Witman et al., 2015). However, long‐term field experiments are constrained by funding, mostly related to experiment deployment and maintenance, and hence can be strongly limited by the lack of permanent financing instruments. This suggests a positive relationship between funding grant and complexity of experimental studies.

Here, we review manipulative field experiments conducted along the SE Pacific coast in order to explore the role of biotic and physical processes influencing coastal community structure and to identify the main limitations related to field experimentation. This study aimed to answer four interconnected questions related to the heterogeneity of ecosystems present along the SE Pacific coast; what have been the ecological processes/mechanisms considered in field experimental studies? Is there a relationship between experimental complexity and the biotic interactions studied? Is there a latitudinal or temporal pattern in the main biotic interactions/processes and habitat types studied? What have been the main constraints for experimentation? Consequently, we examine manipulative experimental studies conducted in the SE Pacific coast from 0°S to 56°S (Ecuador to Chile) published from 1970 to 2016, in this way encompassing ample geographic regions and ecosystems across the Humboldt Current System (Tarazona & Arntz, 2001; Thiel et al., 2007; Vinueza et al., 2014). Specifically, we examined (a) latitudinal trends in the proportion of manipulative experimental studies conducted, (b) the habitat types (rocky, sandy; intertidal, and subtidal) considered for experimentation, (c) the proportion of the main ecological interaction studied (i.e., competition, predation, herbivory, and facilitation) and the physical processes considered. In order to explore the level of complexity in the reported studies, we analyze (d) the taxonomic groups (i.e., classes, orders, and/or species) or focal assemblages studied, estimated (e) the number of treatments and design utilized (random, block, or factorial design) in each study, (f) the spatial–temporal extent of experiments, and finally, to validate the focus on specific processes of each study, we considered (g) the proportion of studies presenting specific hypotheses. We also examine, qualitatively as a source of experimental limitations, the number and sources of funding grants reported by the different studies. We finally discuss challenges and new avenues for future field experimental studies, incorporating different dimensions of anthropogenic impacts on coastal ecosystems.

## 2 METHODS

### 2.1 Literature selection and examination

We used different searching strategies to find manipulative experimental studies published from 1970 to 2016. First we searched specific databases like “Web of Science” (WOS) and “Google Scholar” with specific search terms such as marine ecology*, field experiments*, intertidal*, subtidal*, rocky shore*, sandy shore*, coastal wetlands*, predation*, herbivory*, facilitation*, competition* AND South Pacific coast*, Ecuador*, Peru*, Chile*. Second, complementary to the previous search we explored the main specialized international and local marine ecological journals (see Supporting Information Table S1 in Appendix S1) to which we had access in the time range considered. Finally, we reviewed nondigital documents present in the archives of the Universidad Católica del Norte at Coquimbo (collection from 1970s to the present) of marine science journals. Thus, we also included studies written in Spanish for which no digital documents are available. We then classified the information into different habitat types, that is intertidal, subtidal; rocky, soft bottom or sandy habitat and coastal wetlands, and also year of publication and the latitude where experiments were conducted. In addition, experimental designs considered in the different studies were classified in “random,” “block,” and “factorial”. Independent experiments presented in the same publication were considered as different studies (see Supporting Information Table S1 in Appendix S2). The studies presenting incomplete details or information about how field experiments were performed were not considered for analyses. Twelve studies which conducted field experiments were not considered for further analyses, given they do not provide information of either treatment number or experimental design and/or number of replicates. Many of the studies surveyed had high heterogeneity in the presentation of experimental procedures and results (*n *~ 50), and only a small subset of studies reported means and standard deviation (*n* = 12), which did not allow meta‐analysis (Gurevitch & Hedges, 1999). In our survey, we only considered experimental manipulative studies which utilized a formal experimental design and “manipulated” single or multiple variables (physical, chemical and/or biological) (according to Underwood, 1999). The defining feature of a manipulative experiment is that the different experimental units receive different treatments, and that the assignment of treatments to experimental units is or can be randomized (Hulbert, 1984).

Funding agencies or grants reported by each study were separated into the following categories; national public (government or organizations), local institutions (universities, research centers) and private firms, and international (i.e., public and private agencies or organizations not from Ecuador Peru or Chile). As no study reported the total monetary cost during experimentation, we only made an estimation of the impact of the number of funding agencies reported by different studies and the spatial–temporal extent of experiments. Thus, these analyses should be considered as a first approximation to test the effect of funding on the spatial–temporal extent of field experiments.

### 2.2 Data analysis

To characterize whether there were general spatial and temporal trends in the number of experimental studies conducted, we analyzed the data using quantile regression (Koenker, 2004). Thus, we looked at the relationship between year and number of funding grants, and log (*x* + 1)‐transformed response variables (i.e., spatial extent, elapsed time of experiments) using a wide variety of quantiles (tau). As our dataset is expected to include the entire population of experimental studies (i.e., we have included every relevant publication that exists from 1970 to 2016), we analyze them only qualitatively.

## 3 RESULTS

### 3.1 Regional patterns of manipulative experimental studies conducted in the SE Pacific

**Figure 1:**
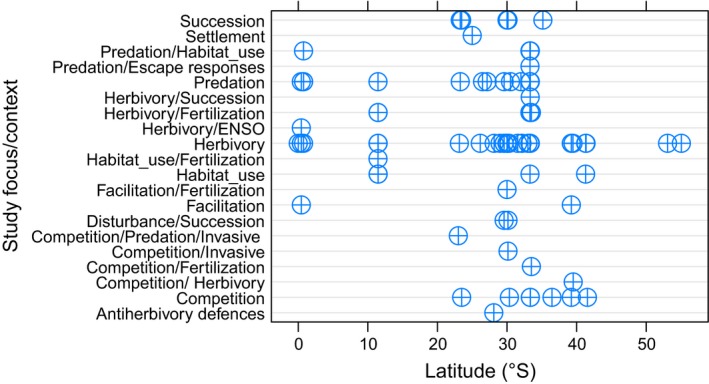
Dot‐plot of the different study foci and contexts (e.g., biotic interaction and environmental processes) considered by field experimental studies along the SE Pacific coast

Of the total number of studies selected (i.e., *N* = 81, of 75 publication, see Supporting Information Appendix S2), about 77% were conducted between 21°S and 40°S in Chilean coasts, while experimental studies conducted along the coast of Peru (~10–15°S) account for about 10.8% of the total of studies surveyed. About 12.2% of the studies were concentrated in the Galápagos Islands in Ecuador (~0–1°S; Figure 1). Most studies were conducted on both intertidal and subtidal rocky shore habitats (52.7% and 35.1% of studies, respectively); the soft bottom habitat has been less used for experimentation (8.1%).

### 3.2 Spatial–temporal analyses of experimental studies

The main focus of manipulative field experimental studies conducted across the SE Pacific was highly heterogeneous, ranging from those dealing with biotic interactions (e.g., competition and predation), or ecological processes (e.g., succession), to mixed studies considering both “top‐down” and “bottom‐up” processes in which both biotic interaction and nutrient levels were manipulated (e.g., “*Herbivory/Fertilization”* in Figure 1). A significant proportion (53%) of studies considered explicit hypotheses in their study design (“yes” vs. “no” hypothesis).

**Figure 2:**
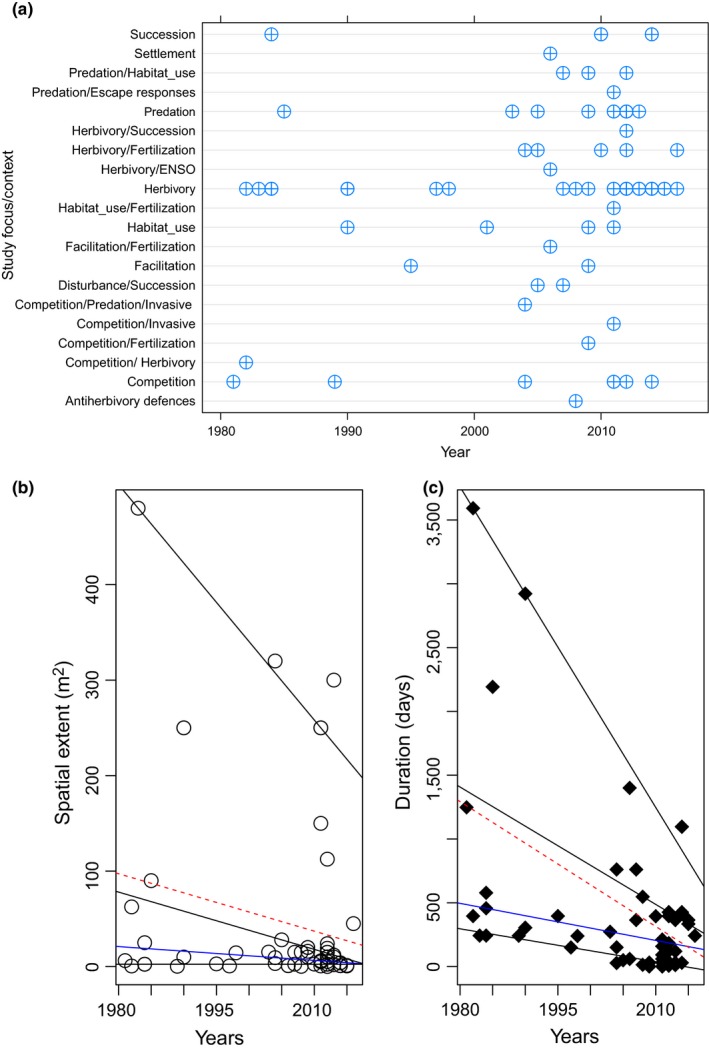
Temporal–spatial patterns of field experiments; (a) Dot‐plot of different manipulative field experiments conducted by year. Scatterplots of the (b) total spatial extent (m) and (c) duration (days) of manipulative field experimental studies. Superimposed on the plot are the {0.25, 0.75, 0.95} quantile regression lines in gray, the median fit in solid blue, and the least squares estimate of the conditional mean function as the dashed (red) line

An increase in the number of manipulative experimental studies by year was detected from 2005 to 2016 (Figure 2a), with more studies concentrated in the band of 2010–2016, suggesting an increase in the proportion of studies in the recent decade. However, analyses showed that spatial extent of field experiments decreased in the upper quantiles (i.e., tau = 0.75; Figure 2b, Supporting Information Table S1a in Appendix S3), while the duration of experiments decreased significantly at the upper and lower quantiles considered (Figure 2c, Supporting Information Table S1a).

### 3.3 Funding and spatial–temporal patterns

**Figure 3:**
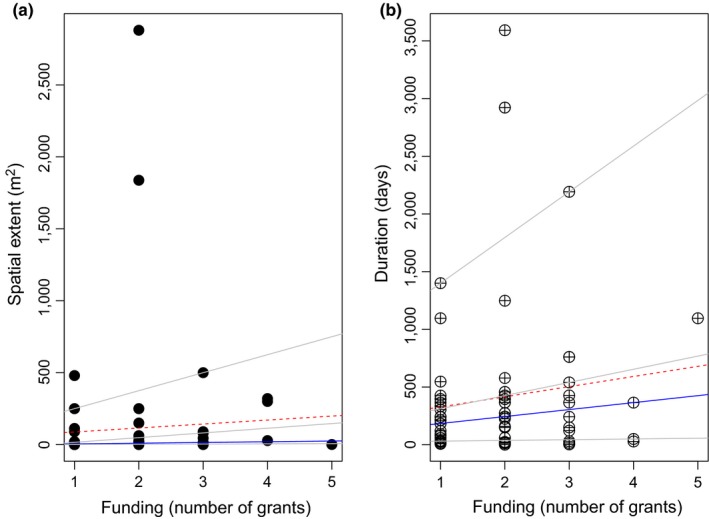
Relationship between duration and spatial extent considered in field experiments and the number of grants reported by the different studies. Superimposed on the plot are the {0.25, 0.75, 0.95} quantile regression lines in gray, the median fit in solid blue, and the least squares estimate of the conditional mean function as the dashed (red) line

The number of funding grants reported by the studies was variable (median = 2 grants per study) and independent of the biotic interaction studied. The spatial extent of experiments conducted was on average less than 10 m^2^, but tended to increase with the number of funding grants in the upper quantile (tau = 0.95, Figure 3a, Supporting Information Table S1b in Appendix S3). The duration of field experiments increased with funding at the median value and at an upper quantile (i.e., tau = 0.75, see blue and gray lines in Figure 3b, respectively). This agrees with the least square estimates of the mean function (blue line in Figure 3b) which showed an increase in experiment duration with the number of funding grants reported.

### 3.4 Manipulation of species interactions and community effects

**Figure 4:**
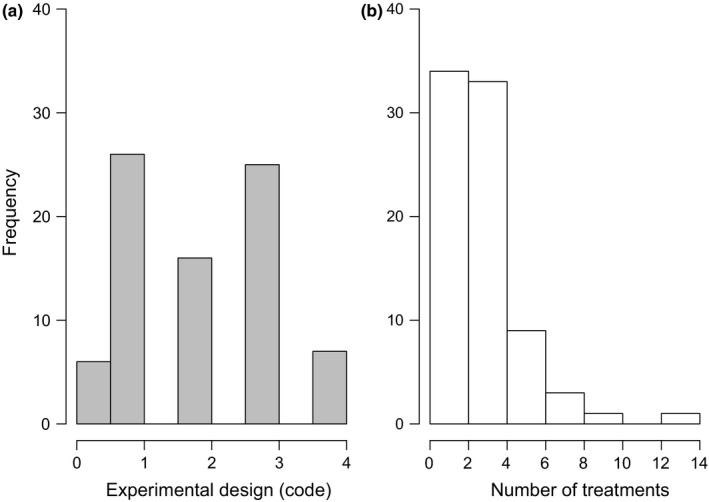
Frequency of experimental studies using (a) specific experimental designs (0: none, 1: random, 2: Nested, 3: Block, 4: Factorial, or mixed designs) and (b) different number of treatments considered in each experiment

The complexity of experimental and treatment designs was variable in the studies considered (Figure 4). Most studies used “random” or “block” experimental designs (codes 1 and 3 in Figure 4a), with a high proportion of studies (>70%) including only two or three treatments (Figure 4b).

**Figure 5:**
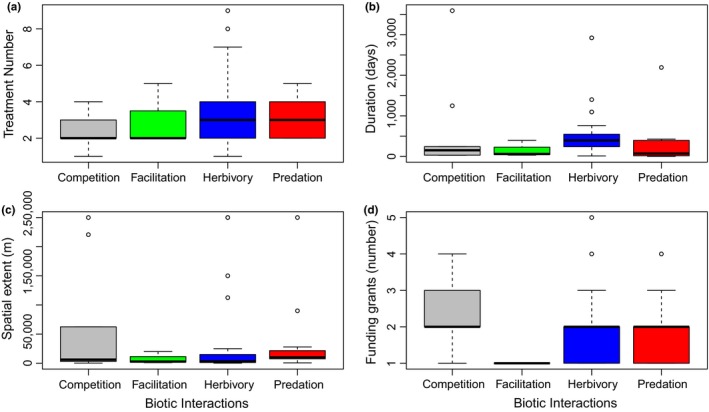
Main factors related to examining the different ecological interactions considered in manipulative field experiments. (a) Number of treatments, (b) Duration, (c) Spatial extent, and (d) Funding grant amount

Most studies were conducted to examine herbivore effects on algae (i.e., herbivory; 45.3%), while studies dealing with predators account for 14.0% and competition studies account for 12.0% of the total. Studies dealing with species facilitation or positive interactions account for less than 4.0% of the total of studies considered. Only three experimental studies dealt explicitly with invasive species in the context of predation or competition (i.e., biotic resistance; see Figure 1). Studies dealing with the main biotic interactions used on average 2–3 treatments (e.g., “consumer excluded,” “consumer present”) (Figure 5a). Herbivory studies showed an ample range of treatments (from 1 to 9 treatments) compared to the other biotic interactions considered (Figure 5a). Marked differences in the distribution of elapsed time of experiments were observed between predation and herbivory studies (Figure 5b). No differences in spatial extent or funding grant number were observed among the biotic interactions considered (Figure 5c, and see Figure 5d, respectively), suggesting that these factors do not explain the differences in the number of studies considering a specific biotic interaction.

**Figure 6:**
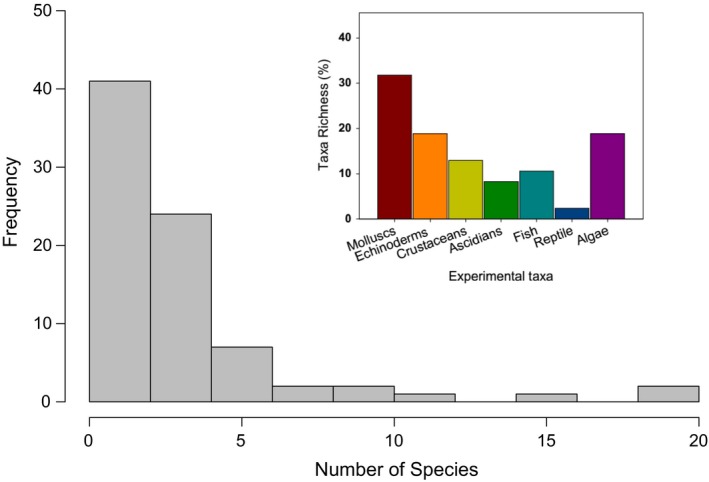
Histogram of the frequency of studies considering different numbers of species. Insert; Percentage of taxa richness (i.e., focal species number within each group) considered in manipulative experimental studies

Most experimental studies considered a small number of interacting species in the experiments, which oscillated between 1 and 4 species (Figure 6). Only a very small set of studies considered explicitly more than 10 species in their designs (Figure 6) using open experiments, with complete removal of consumers from areas of 5 m^2^ or more. However, most of these studies did not use specific control of species abundances. Mollusks, echinoderms, and algae were the most frequent focal taxa considered in the different studies, accounting for 31.7%, 19.3%, and 19.5% of the total of species considered, respectively (see insert in Figure 6).

## 4 DISCUSSION

### 4.1 Regional patterns of manipulative field experimental studies conducted in the SE Pacific

Over the past three decades, the ecology of the diverse and unique species assemblages that inhabit the tropical, subtropical, and temperate Pacific shores of South America (0°S to 42°S) have received increasing scientific attention (Thiel et al., 2007). Coastal regions present from 0° to 42°S are influenced by the Humboldt Current System (HCS) and are strongly interconnected by large‐scale processes like “El Niño” events (Thiel et al., 2007; Vinueza et al., 2014). Experimental studies performed along this gradient have contributed to fundamental and applied knowledge about management of diverse marine coastal ecosystems. In this review, we have described the diversity and complexity of field experimental studies conducted along the SE Pacific coast. In our review of the literature from 1970 to 2016, we found high heterogeneity of experimental studies along the SE Pacific coast, and high variation of temporal and spatial scales of experimentation (i.e., their complexity). Most studies contribute to understand the importance of bottom‐up and top‐down processes along the SE Pacific coast. Subtropical and temperate systems from 20°S to 40°S have been the most studied, but with notable experimental studies conducted in intertidal and subtidal habitats in the Galápagos Islands (e.g., Brandt, Witman, & Chiriboga, 2012; Irving & Witman, 2009; Vinueza, Branch, Branch, & Bustamante, 2006; Vinueza et al., 2014). Although our study encompasses an ample geographic gradient, we did not consider emergent experimental studies conducted in the Antarctic Peninsula (e.g., Segovia‐Rivera & Valdivia, 2016) which examined the main processes structuring coastal communities in extreme environmental conditions.

Our findings reveal that most manipulative experimental studies have been conducted from 18°S to 45°S in Chile, most of which (e.g., Castilla & Durán, 1985; Jara & Moreno, 1984; Moreno, Sutherland, & Jara, 1984; Paine, Castilla, & Cancino, 1985) have resulted in applied knowledge to manage ecosystems under intense human harvesting and fisheries (Castilla, 2000; Castilla & Fernandez, 1998; Fernández et al., 2000; Gelcich et al., 2010; Vasquez & Buschmann, 1997). It seems that the strong emphasis in field experimental‐based methods developed during 1970s and 1980s (Castilla, 2000) has motivated a new generation of experimental ecologists in Chile, which may explain the higher number of experimental studies found in the last decade. An important number of studies have been conducted recently (2008 to present) in Peru (around 10°S; Hidalgo et al., 2008; Firstater et al., 2010; Firstater, Hidalgo, Lomovasky, & Iribarne, 2012), which could also motivate the development of marine field experimental research at these latitudes. Interestingly, most field manipulative studies conducted in Ecuador (Galápagos Islands) and Peru report international funding and/or were conducted by non‐native researchers (i.e., reporting foreign associated institutions). It could be of interest to examine further the development of experimental ecology in these latitudes, and how basic ecological knowledge can be effectively translated into management plans and/or conservation priorities as in the Galápagos Islands (e.g., Calvopiña, Chamorro, Cruz, Tapia, & Izurieta, 2015).

### 4.2 Spatial–temporal complexity of experimental research

In a general review of the advances in marine experimental development, Witman et al. (Witman et al., 2015) examined the historical progress in the field in incorporating higher levels of complexity in manipulative experimental designs. Although field experimental studies along the SE Pacific have been increasing in the last decade, especially in rocky intertidal habitats, we found that most studies considered a short time (25–200 days), and small spatial extent (~20 m^2^) in their design, which is in agreement with results of Witman et al. (2015) which considered a global set of data. However, we found that field experimental studies considered a small number of treatments (2–3) and focal species (2–4 species), which is contrary to the general patterns observed previously (Witman et al., 2015). Either abundance or economic interest of focal species, or both, could explain the reduced number of factors and species considered. Alternatively, limitation imposed by monetary budgets may have constrained the spatial and temporal extent and the number of factors included in field experiments. Our results suggest that longer studies could be most limited by number of grants involved in the study, but they seem not to influence the type of biotic interaction studied.

### 4.3 Biotic interactions and experimental design

Our study showed that 51.6% of experimental studies incorporated only 2–3 treatments in their design, which were related to examine the combined effect of 2–3 different factors like nutrients and consumers, or predation effect and competition. For example, some experimental studies considered either fertilization of experimental arenas (Firstater et al., 2012) or a set of localities with different nutrient (e.g., upwelling regime) conditions as crossed factors with consumer effect (Nielsen & Navarrete, 2004). Results of these studies showed how the specific environmental context could influence/modulate the intensity of species interaction, which is a matter of broad interest in different ecological systems (Chamberlain, Bronstein, & Rudgers, 2014). Studies which evaluated concurrently the effect of competition and predation on intertidal communities of habitat‐forming species (e.g., ascidians; Caro, Guiñez, Ortiz, & Castilla, 2011; Castilla, Lagos, & Cerda, 2004) showed the importance of considering concurrently these ecological processes and their joint role in shaping intertidal structure (see Chesson & Kuang, 2008). Only a few experimental attempts have been made considering the role of positive interactions (i.e., facilitation and mutualism) (Irving & Witman, 2009), or the effects of invasive species on native species and biotic resistance (Caro et al., 2011; Dumont, Gaymer, & Thiel, 2011) along the SE Pacific coast. It is not clear, however, whether lack of studies on this topic in the SE Pacific coast is related to the low frequency of these interactions or if there is still a prevailing view among ecologists that negative interactions (predation and competition) are the main processes determining species distributions and abundance (see Bruno, Stachowicz, & Bertness, 2003; Bulleri, 2009 for discussion). Further studies are thus needed to investigate these aspects and to determine the role of positive interactions influencing species coexistence and the structure of coastal marine communities in the SE Pacific.

Studies on consumer–resource interactions (especially herbivore–alga interaction) were the most frequent along the range considered in our investigation. Most studies were based on temperate systems and rocky intertidal habitats, which might reflect the proportion of studies considering mollusks and sea urchins as focal species in field experiments. These invertebrate groups are dominant in temperate systems along the SE Pacific coast, and most species are feasible for field experimentation. Herbivory experiments commonly included two to three treatments, in which herbivores are “excluded” (i.e., removed from parcels or plots) and/or “enclosed” in different experimental arenas, followed by “procedural control” (i.e., partial fences or partial antifouling paint; Aguilera & Navarrete, 2007; Nielsen & Navarrete, 2004). A special case is the study of Vinueza et al. (Vinueza et al., 2006; and see also Vinueza et al., 2014), which considered the individual and collective impact (i.e., mixed consumption effects) of different tropical herbivore taxa (reptiles, crabs, and mollusks) on community structure (algae and invertebrates) before and after an El Niño event in the Galápagos Island. This study provided important information about how large‐scale episodic processes can regulate local‐scale ecological interaction and community structure. Of special mention are the studies considering the combined impact of nutrient levels, plant competition, and domestic cattle grazing on coastal saltmarsh plant composition (Fariña, He, Silliman, & Bertness, 2016; Fariña, Silliman, & Bertness, 2009). It is worth noting that these are the only field manipulative experimental studies conducted (or published) on coastal wetlands in the range considered.

### 4.4 Large‐scale pattern in experimental studies: contribution and limitations

Given the ample biogeographic regions present along the SE Pacific coast, experimental studies dealing with herbivore–alga interaction could contribute greatly to examine latitudinal trends in the role of herbivore on alga composition (Lubchenco & Gaines, 1981). It should be noted, however, that although most experimental studies surveyed focused on herbivory, only a small fraction of them have been considered by previous specific reviews (Poore et al., 2012). Likely, some studies were not considered because they are written in Spanish instead of English and/or did not have digital access. In the present review, we considered all of them.

Marine herbivores are expected to have a primary role in tropical versus temperate latitudes (Burkepile & Hay, 2006). However, other studies suggest that herbivores can have a strong effect in temperate latitudes and a weaker effect in the tropics (Poore et al., 2012; Vinueza et al., 2006). Manipulative field experiments considering different upwelling or nutrient conditions in the range considered in this review showed that herbivory can vary according to productivity levels (Firstater et al., 2012; Hidalgo et al., 2008; Nielsen & Navarrete, 2004) and microhabitat use (Firstater et al., 2010) with no marked trends in latitudinal patterns, and thus, the role of top‐down versus bottom‐up drivers would be context‐dependent (see also Vinueza et al., 2014). This may be due to differences in oceanographic regimes and consumer composition at different localities, and field experimental studies should progress further to incorporate this variability in their designs. Clearly, one of the limitations is related to the technical feasibility to conduct and maintain field experiments at different localities. Another limitation is the monetary cost associated with maintaining spatially and temporally large field experiments, as suggested above. We found that a higher number of funding grants reported was related to an increase in the duration of manipulative experimental studies. It should be noted, however, that the number of funding grants may not reflect the scale of economic budgets available in each research project but at least suggest they have a primary role in the spatial and temporal extent of field experiments. Future studies should analyze, for example, the average monetary costs associated with implementing and monitoring field experiments at a temporal scale large enough to capture important ecological processes like population dynamics or community succession. This could shed light into how monetary budgets affect scientific progress in this research field.

Many natural and anthropogenic modifications of ecosystems are taking place along the SE Pacific coast; clear cooling trends (i.e., negative temperature anomalies) have been observed across the Humboldt Current System (Lima & Wethey, 2012; Rykaczewski et al., 2015; Wang, Gouhier, Menge, & Ganguly, 2015), and human harvesting in different trophic groups is intensifying (e.g., consumers; Godoy, Gelcich, Vásquez, & Castilla, 2016; kelps; Krumhansl et al., 2016). In addition, construction of artificial coastal infrastructures is increasing in some coasts (e.g., Chile; Aguilera, 2017), and different urbanization processes associated with increased levels of marine pollutants (Bravo et al., 2009; Fariña, Castilla, & Ojeda, 2003; Lancellotti & Stotz, 2004; Thiel et al., 2011). Non‐native species proliferation (Neill, Alcalde, Faugeron, Navarrete, & Correa, 2006; Villaseñor‐Parada, Pauchard, & Macaya, 2017) has the potential to harm intertidal, subtidal, and coastal wetland species biodiversity along the SE Pacific coasts.

In this review, we found some experimental studies that deal directly with determining the impact of human harvesting and its propagation to community structure in intertidal rocky shore systems (e.g., kelp harvesting; Oróstica, Aguilera, Donoso, Vásquez, & Broitman, 2014; “human‐exclusion” experiments; Moreno, 2001; Castilla & Durán, 1985; Castilla, 2000; Moreno et al., 1984). No field experimental studies, however, deal with pollution in coastal habitats. Although some studies conducted experiments in highly polluted areas (e.g., Correa, Ramírez, De La Harpe, Román, & Rivera, 2000), the hypotheses and main goals of these studies did not deal directly with impacts of pollution on biotic species interactions. Manipulative field experiments on this topic could enhance our level of knowledge about the impacts of pollution on both trophic and nontrophic interactions affecting the rich topology of coastal ecosystem interaction webs (e.g., see Kéfi, Miele, Wieters, Navarrete, & Berlow, 2016). Similarly, as the proliferation of artificial infrastructures or man‐made structures is replacing an important proportion of natural habitats in the SE Pacific coast (e.g., 18°S–42°S; Aguilera, 2017), incorporation of field experiments in these habitats may be useful to forecast loss of species interactions or functional diversity. In fact, coastal artificial infrastructures can be viewed as “natural experiments” (Burt, Sale, & Bartholomew, 2011; Feary, Burt, & Bartholomew, 2011), where we can observe the dynamics of local communities in space and time and species adaptation to novel substrates (Bulleri & Chapman, 2010). However, manipulative studies adapting specific treatment and experimental designs are needed to determine the impact of artificial infrastructures on biotic interactions (e.g., see Ferrario, Iveša, Jaklin, Perkol‐Finkel, & Airoldi, 2016; Klein, Underwood, & Chapman, 2011) and to examine the potential for ecological rehabilitation of habitats degraded by human intervention. Therefore, a “comparative‐experimental approach” (Menge, Berlow, Blanchette, Navarrete, & Yamada, 1994; Paine, 2010) consisting of repeating similar manipulations at large geographic scales (e.g., see Rodemann & Brandl, 2017), could shed light into how to improve biotic assemblage functional structure by eco‐engineering procedures and for an integrated ecosystem management (Chapman & Underwood, 2011; Firth et al., 2016; Strain et al., 2017).

## 5 CONCLUDING REMARKS

Diverse manipulative field experiments have been conducted in the SE Pacific, which contribute directly to applied knowledge to manage and conserve natural coastal ecosystems in the face of rapid environmental shift trends and modification of biotic interactions (Ling et al., 2014; Mieszkowska, Broitman, Helmuth, & Blanchette, 2008; Wernberg et al., 2012). Large areas of the coast along the Humboldt Current System are currently experiencing increasing levels of pollution and urbanization, and some emergent economies plan to expand coastal development (mining, aquaculture, and tourism). This means the risk of damage or degradation of coastal ecosystems due to anthropogenic actions is imminent, as in other coastal ecosystems (Defeo et al., 2009; Gittman et al., 2015; Waltham & Sheaves, 2015). Marine ecosystem vulnerability to anthropogenic impacts can be approached directly by changing from purely mensurative to a field experimental manipulative‐mensurative approach to cope with increasing harvesting and pollution hazards.

## AUTHOR CONTRIBUTIONS

MAA involved in the conceptualization, literature search, analyses, writing, and editing the article. JD and IJP involved in writing and literature search.

## DATA ACCESSIBILITY

All data are entirely accessible and available in Excel format in Supporting Information Appendix S2.
