# Supplementary material for: Heterogeneity of ecological patterns, processes, and funding of marine manipulative field experiments conducted in Southeastern Pacific coastal ecosystems
Source: Ecol Evol. 2018 Jul 25;8(16):8627–38. doi: 10.1002/ece3.4371 (PMC6145005; doi:10.1002/ece3.4371)
Supplement: Supplementary file 1 [file ECE3-8-8627-s001.docx]

**Appendix S1**

**Table S1**. List of specialized marine scientific journals considered in the review. Each journal was individually searched complementary to the specific database-searching conducted (see Methods section). Search of individual journals considered the specific search terms; field experiments*, intertidal*, subtidal*, rocky shore*, sandy shore*, AND South Pacific coast*, Ecuador*, Perú*, Chile*

**a)**

| **Journal Name** | **Period of available collection** |
| --- | --- |
| ***Scientific Journals which include articles exclusively written in English*** | |
| *Marine Ecology Progress Series* | *1979-2016* |
| *Journal of Experimental Marine Biology and Ecology* | *1970-2016* |
| *Marine Ecology* | *1996-2016* |
| *Hydrobiology* | *1993-2016* |
| *Marine Biology* | *1970-2016* |
| *Oceanography and Marine Biology An Annual Review* | *1991-2016* |
| *Limnology and Oceanography* | *1979-2016* |
| *Marine Environmental Research* | *1999-2016* |
| *Journal of the Marine Biological Association of UK* | *1970-2016* |
| *Journal of Sea Research* | *1996-2016* |
| *Helgoland Marine Research* | *1970-2016* |
|  |  |
|  |  |
| *Marine and Freshwater Behaviour and Physiology* | *1970-2016* |
| *Marine Pollution* | *1999-2016* |
| *Aquatic Biology* | *2007-2018* |
| ***Scientific Journals which also include articles written in Spanish*** |  |
| *Scientia Marina* | *1989-2016* |
| *Gayana* | *2000-2016* |
| *Revista de Biología Marina y Oceanografía* | *1970-2016* |
| *Revista Peruana de Biología* | *1974-2016* |
| *Revista Chilena de Historia Natural* | *1983-2016* |
| *Latin American Journal of Aquatic Research (LAJAR)* | *1994-2016* |
