# Supplementary material for: Heterogeneity of ecological patterns, processes, and funding of marine manipulative field experiments conducted in Southeastern Pacific coastal ecosystems
Source: Ecol Evol. 2018 Jul 25;8(16):8627–38. doi: 10.1002/ece3.4371 (PMC6145005; doi:10.1002/ece3.4371)
Supplement: Supplementary file 3 [file ECE3-8-8627-s003.docx]

| **a) Time (year of publication) on variables** | | | | | |
| --- | --- | --- | --- | --- | --- |
| **Variable** | **tau** | **coeff** | **Lower bd** | **Upper bd** |  |
| Spatial | 0.25 | -0.04615 | -0.06545 | 0.0381 |  |
| Spatial | 0.5 | -0.01387 | -0.819 | 0.2645 |  |
| Spatial | 0.75 | **-2.4** | **-9.94** | **-0.2095** |  |
| Spatial | 0.95 | -8.2143 | -7.6325 | 6.4687 |  |
| Duration | 0.25 | **-9.58333** | **-13.39113** | **-7.0362** |  |
| Duration | 0.5 | **-8.1333** | **-15.824** | **-4.5478** |  |
| Duration | 0.75 | **-26.516** | **-106.505** | **-46.0067** |  |
| Duration | 0.95 | **-95.125** | **-117.729** | **-77.8514** |  |
|  |  |  |  |  |  |
| **b) Funds (grants number) on variables** | | | | | |
| **Variable** | **tau** | **coeff** | **Lower bd** | **Upper bd** |  |
| Spatial | 0.25 | 0.1125 | -0.3927 | 1.9221 |  |
| Spatial | 0.5 | 6.1000 | -2.1988 | 10.4702 |  |
| Spatial | 0.75 | 10.0000 | -2.3646 | 41.1771 |  |
| Spatial | 0.95 | **23.3333** | **17.6614** | **66.2360** |  |
| Duration | 0.25 | 59.5000 | -14.7262 | 119.9204 |  |
| Duration | 0.5 | **122.000** | **40.2975** | **248.3229** |  |
| Duration | 0.75 | **197.7500** | **30.5796** | **259.3153** |  |
| Duration | 0.95 | 1522.000 | -570.3403 | 3081.874 |  |

**S1Table. Results of quantile regression analysis of a) time (year of publication) and b) Funding grants, on spatial extent and duration of field experiments. Tau values represent the quantile of data used for each regression coefficient and lower (2.5%) and upper (97.5%). intervals.**
